# Forensic Aspects of Designer LSD Analogs Identification by GC–MS (EI) and UV Spectroscopy

**DOI:** 10.3390/molecules29235717

**Published:** 2024-12-04

**Authors:** Kaja Tusiewicz, Olga Wachełko, Marcin Zawadzki, Paweł Szpot

**Affiliations:** 1 Institute of Toxicology Research, 45 Kasztanowa Street, 55-093 Borowa, Poland; 2 Department of Social Sciences and Infectious Diseases, Faculty of Medicine, Wroclaw University of Science and Technology, 27 Wybrzeże Wyspiańskiego, 50-370 Wrocław, Poland; 3 Department of Forensic Medicine, Wroclaw Medical University, 4 J. Mikulicza-Radeckiego Street, 50-345 Wroclaw, Poland

**Keywords:** designer LSD analogs, new psychoactive substances, GC–EI-MS, UV spectroscopy, drugs identification, isomers separation

## Abstract

Lysergic acid diethylamide (LSD) analogs, often referred to as new psychoactive substances, are synthesized to mimic controlled substances while evading drug regulations. This study emphasizes the challenges of identifying these compounds, particularly their isomeric forms. Gas chromatography–mass spectrometry (GC–MS) and UV spectroscopy were employed to analyze 13 LSD analogs. The effects of different solvents on the detection of these analogs were analyzed, demonstrating that solvents like diethyl ether, *tert*-butyl methyl ether, dichloromethane and acetone provided the best sensitivity and stability. Methanol, on the other hand, causes alcoholysis of many LSD analogs, which may lead to false results. Additionally, effective chromatographic separation of isomers was established, including LSD, MiPLA, LAMPA, 1P-LSD and 1P-MiPLA, as well as 1cP-LSD and 1cP-MiPLA, which is crucial for accurate identification. The elution order of the determined compounds with the use of developed chromatographic method was as follows: LSD, MiPLA, LAMPA, AL-LAD, LSZ, 2-Br-LSD, ALD-52, 1P-LSD, 1P-MiPLA, 1B-LSD, 1V-LSD, 1cP-LSD and 1cP-MiPLA. Differences in ion ratios observed in mass spectrometry (MS) were also analyzed to distinguish between closely related compounds. Several key ions for LSD analogs were able to be identified, including 221, 208, 207, 196, 194, 192, 181, 167, 154, 152 and 128 *m*/*z*. In analogs with an *N*-diethyl group (or variants like *N*-methyl-propyl in LAMPA or *N*-methyl-isopropyl in MiPLA), mass spectra showed fragments 100, 72 and 58 *m*/*z*. For LSZ, the cyclic group at R1 produces ions 98 and 70 *m*/*z*. Analogs with an *N*6 allyl group (e.g., AL-LAD) show a characteristic ion 247 *m*/*z*. This method allows for the correct differentiation of structural isomers based on their unique ion fragmentation patterns and relative intensities. UV spectroscopy was used as a supplementary tool for screening, though it has limitations in analyzing complex mixtures. This work contributes to the forensic identification of designer LSD analogs, ensuring reliable detection for legal and toxicological investigations.

## 1. Introduction

Lysergic acid diethylamide (LSD) is a potent psychedelic known for its profound effects on consciousness. Recently, the recreational use of designer LSD analogs has increased, driven in part by their accessibility through the dark web and other online platforms. New psychoactive substances (NPSs) are structurally modified to mimic controlled substances while evading drug regulations, creating legal loopholes. The rapid synthesis of new NPSs (often following the prohibition of existing compounds) perpetuates regulatory challenges and maintains legal ambiguities. In recent decades, there has been a notable resurgence in the synthesis and distribution of LSD analogs, often termed “designer psychedelics” or “research chemicals” [1,2,3]. These compounds are structurally related to LSD and typically involve slight modifications to the lysergamide backbone, resulting in substances that can mimic, enhance or even subtly alter the effects of LSD. Moreover, compounds such as ALD-52 or 1P-LSD appeared on the market initially to circumvent regulations while simultaneously being “prodrugs” [4].

The growing proliferation of designer LSD analogs presents considerable challenges. These substances often evade current drug regulations, complicating enforcement efforts and increasing public health risks. The absence of thorough safety data for many of these analogs further intensifies the danger, as users may encounter unknown or unpredictable effects. Several international initiatives have been crucial in monitoring the proliferation of NPSs. The United Nations Office on Drugs and Crime (UNODC) has identified over 1220 NPSs globally, with a substantial proportion comprising synthetic cannabinoids, cathinones and lysergamides, including designer analogs of LSD [5]. From 2010 to 2017, the total number of LSD seizures has doubled, though the amount seized has varied over time [6]. This may be due to the fact that the monitoring of hallucinogenic drug seizures is not consistent across Europe, and the data come from various monitoring systems, but it is limited, fragmented and challenging to generalize [7]. This highlights the issue that these seizures are not analyzed in a standardized, unified manner.

In toxicological examinations, special attention must be given to the specific differentiation of isomeric compounds of new psychoactive substances (e.g., MiPLA and LAMPA or 1P-MiPLA and 1P-LSD). Isomers can exhibit markedly different pharmacological properties, including potency, efficacy and safety, making it crucial to predict their effects on users. Additionally, legal definitions of controlled substances often hinge on specific chemical structures, so accurately identifying isomers is vital in enforcing drug regulations effectively. The presence of isomers can create loopholes that allow substances to evade regulation, underscoring the importance of proper classification. In forensic toxicology, identifying the specific isomer involved in an overdose or present in drug samples collected from a crime scene is critical for elucidating the circumstances surrounding the incident and facilitating criminal investigations. The significance of this is demonstrated in cases, including those involving LSD analogs, in which a drug sold as one analog ultimately turned out to contain a completely different LSD analog [8,9,10]. This illustrates how important it is in forensic toxicology to determine the specific analog involved, as this has legal implications and is crucial in prosecutorial cases.

However, a number of factors can affect the final result of an analysis by gas chromatography coupled with mass spectrometry (GC–MS), which is the most commonly used method for analyzing seized drug samples. It is necessary to consider adjusting the appropriate temperature gradient to separate the isomers, as well as selecting the right solvent to avoid situations in which some LSD analogs undergo alcoholysis [11,12] by breaking the amide bond and forming other analogs (such as LSD or MiPLA), which leads to false analysis results. Additionally, consideration should be given to newly emerging, not yet investigated analogs that may appear on the market. For the identification of such compounds, a fragmentation pathway in mass spectrometry may be helpful, as libraries of spectra may not be available for these new compounds. It is noteworthy that this issue is also problematic in general NPS screening, and the rapid rise and evolving nature of NPSs pose challenges for forensic drug testing laboratories. Degradation in GC–MS analysis has also been observed with the NBOH drug, where alcohol solvents enhanced degradation via nucleophilic substitution, yielding 2C-series compounds [13]. Phenibut and ethylphenidate also showed similar degradation [14]. What is more, QUPIC (PB-22) degrades into multiple products when dissolved in methanol or ethanol but shows less degradation in non-alcoholic solvents like acetone or chloroform [15]. These cases underscore the limitations of GC–MS, especially when using alcohol-based solvents.

**Figure 1:**
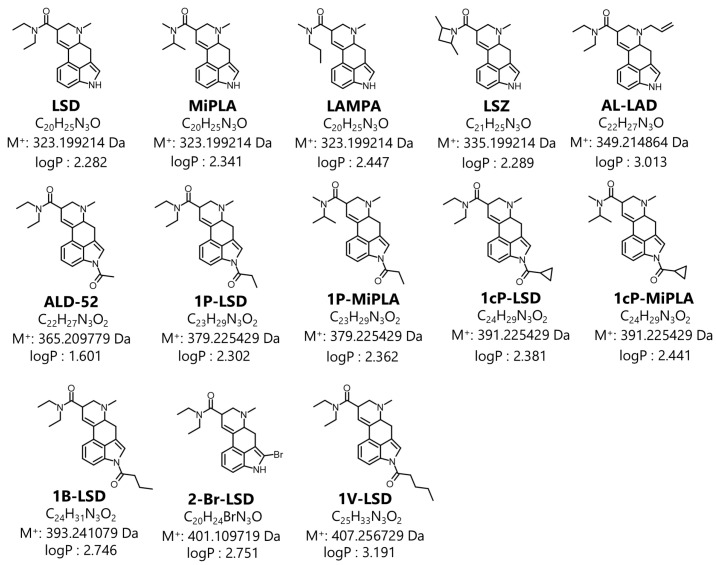
Structures of designer LSD analogs analyzed in the presented paper. The logP values were obtained with the use of Chemicalize software (https://chemicalize.com).

Thus, the aim of this study was to address the aforementioned issues related to the analysis of LSD analogs for forensic toxicology purposes. Thirteen LSD analogs were analyzed (Figure 1). GC–MS analysis conditions, fragmentation pathways and solvent effects have been extensively studied and described. In addition, UV spectra of the analyzed compounds, which can also serve as additional confirmation of the presence of a given compound in a seized drug sample, are presented and discussed. Furthermore, these spectra may also be useful in developing countries where facilities such as GC–MS are unavailable.

## 2. Results and Discussion

### 2.1. Mass Spectra (EI-MS) and Fragmentation Pathways

**Figure 2:**
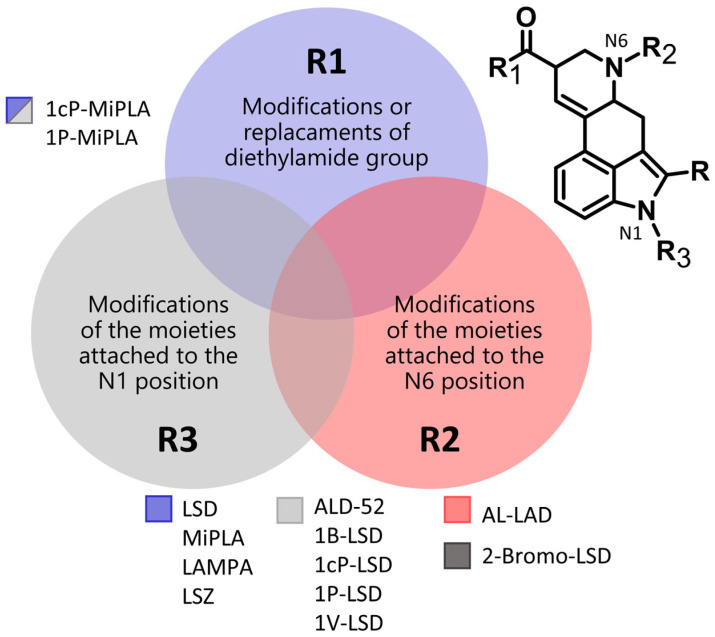
Differences in the chemical structure of designer LSD analogs.

**Figure 3:**
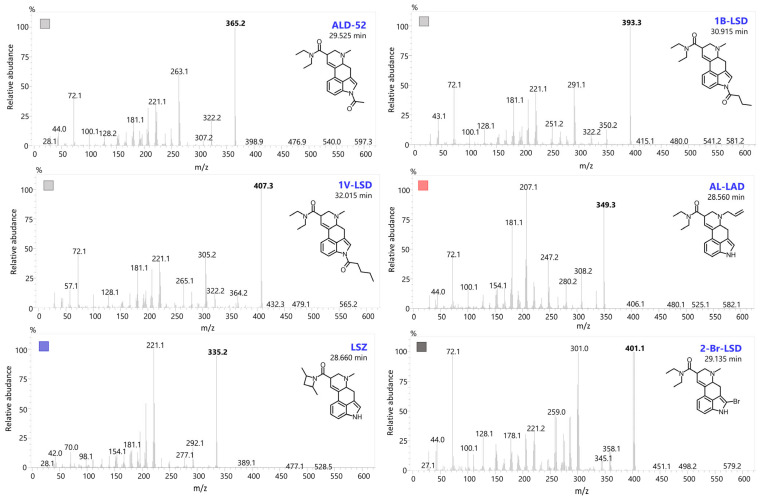
EI-MS spectra in scan mode of six designer LSD analogs: ALD-52, 1B-LSD, 1V-LSD, AL-LAD, LSZ and 2-Bromo-LSD.

**Figure 4:**
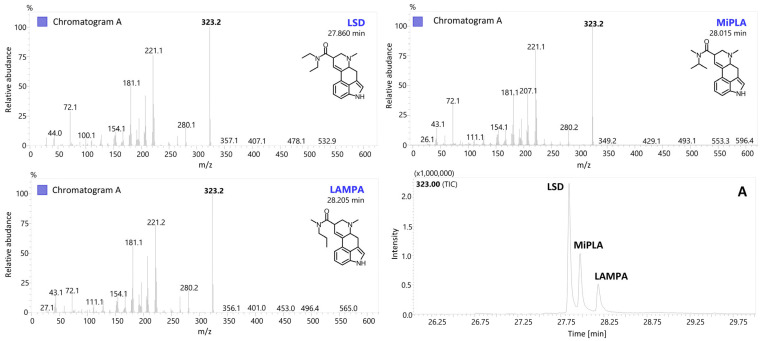
EI-MS spectra in scan mode of LSD, MiPLA and LAMPA with chromatographic separation (chromatogram A).

**Figure 5:**
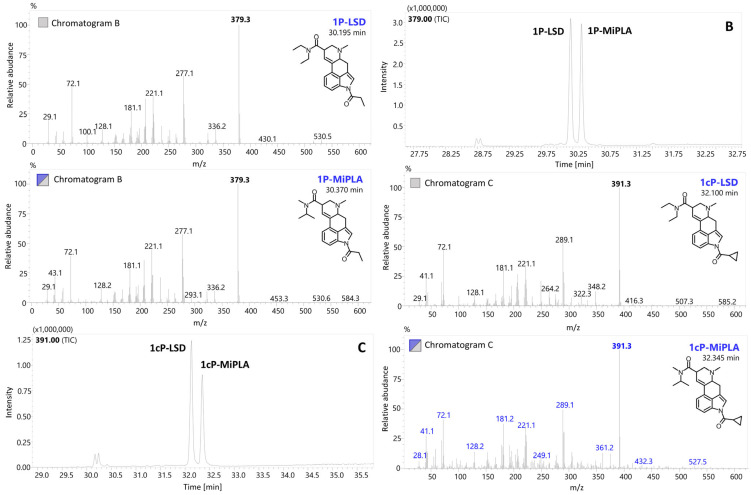
EI-MS spectra in scan mode of two sets of structural isomers with chromatographic separation: 1P-LSD and 1P-MiPLA (chromatogram B) as well as 1cP-LSD and 1cP-MiPLA (chromatogram C).

Typically, EI-MS is used in the analysis of seized drugs to generate a distinctive mass spectrum for a compound, allowing unknown substances to be identified by comparing them with a mass spectral library or known reference standards. The structural details obtained from EI-MS are particularly valuable for detecting novel compounds, especially when they involve only slight modifications to known substances. The designer LSD analogs characterized in this paper can be divided into three main groups based on the types of substituents (Figure 2). Variations in the chemical structures of these analogs can involve slight modifications to the lysergamide backbone, such as substitutions at the *N*1 position (e.g., ALD-52, 1B-LSD, 1cP-LSD, 1cP-MiPLA, 1P-LSD, 1P-MiPLA and 1V-LSD), the *N*6 position (e.g., AL-LAD) or modifications or replacements of the diethylamide moiety (e.g., LAMPA, MiPLA and LSZ). For each analyzed compound, the EI-MS spectrum (70 eV) was collected and is presented in Figure 3, Figure 4 and Figure 5.

**Figure 6:**
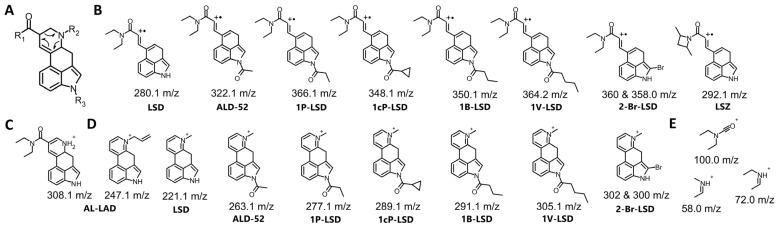
Structures of selected characteristic EI-MS ions for different LSD analogs.

The fragmentation pattern of LSD was first proposed by Nigam et al. in 1969 [16]. In their work, they identified the structures of characteristic fragments for LSD, including 207 *m*/*z*, 167 *m*/*z* and 154 *m*/*z*. These fragment structures are also applicable to a wide range of LSD analogs, making them universally relevant for the identification of these compounds. In the case of most LSD analogs (with the exception of 2-Br-LSD), several characteristic key ions can be identified, including 221, 208, 207, 196, 194, 192, 181, 167, 154, 152 and 128 *m*/*z*. Structures of selected characteristic EI-MS ions for different LSD analogs are presented in Figure 6. In analogs containing an *N*-diethyl moiety (or its variations, such as *N*-methyl-propyl in LAMPA or *N*-methyl-isopropyl in MiPLA), fragments with *m*/*z* 100, 72 and 58 are visible in the mass spectra. When analyzing LSZ, it can be observed that, due to the cyclic moiety at R1, other ions are produced during ionization, notably at 98 and 70 *m*/*z*. Analogs with an allyl group (e.g., AL-LAD) attached to *N*6 give the characteristic ion at 247 *m*/*z*. For 2-Br-LSD, fragments are visible that are increased by the mass of bromine, which does not detach from the molecule immediately. For this reason, many fragment ions, as well as the precursor itself, show a characteristic isotopic pattern. Bromine-containing compounds’ MS spectra are distinctive because of two naturally occurring stable isotopes, Br-79 (78.918) and Br-81 (80.916), which exist in almost equal proportions. This results in a distinctive pattern that can aid in detecting bromine within a molecule. In mass spectrometry, bromine is easily recognized by its unique 1:1 peak ratio and the 2 Da mass difference between the peaks. Thus, in 2-Br-LSD, the visible ions are: 302 and 300 *m*/*z*, 286 and 284 *m*/*z* and 260 and 258 *m*/*z*.

**Figure 7:**
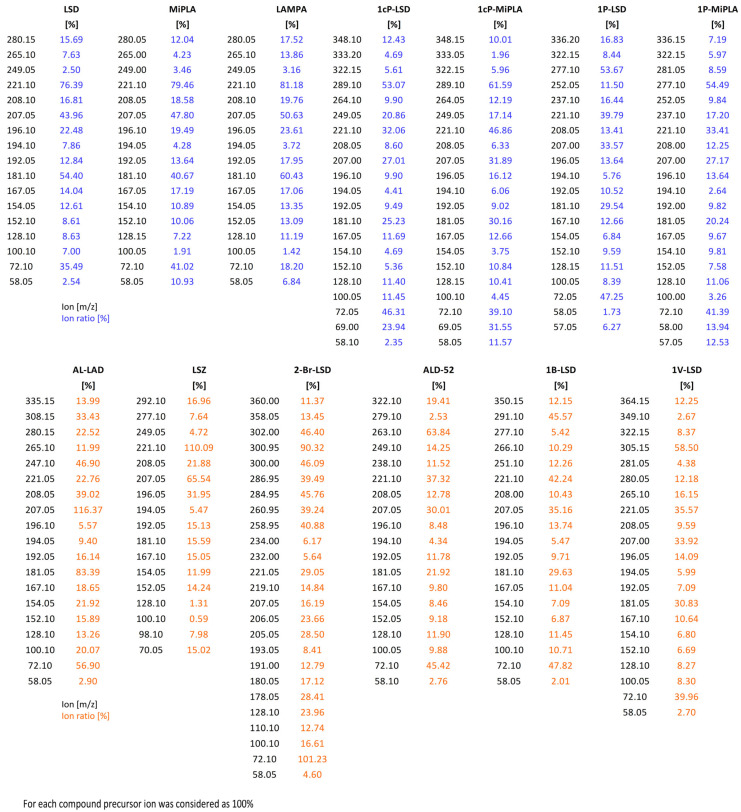
Ion ratio in EI-MS analysis of 13 designer LSD analogs.

Brandt et al. [17] found that isomeric analogs (LSD, MiPLA and LAMPA) differ in the intensity of the iminium ion at 72 *m*/*z* during tandem mass spectrometry (MS/MS) experiments, in which this ion was subjected to further collision-induced dissociation (CID) analysis. It is worth noting, however, that even in standard electron ionization (EI) mass spectra at 70 eV, the ratio of specific ions can be helpful in distinguishing isomers, as demonstrated in their paper. In many forensic laboratories, gas chromatography–mass spectrometry (GC–MS) with a single quadrupole is the gold standard for the rapid identification of illicit drugs in seized samples. Under these conditions, performing product ion scan experiments (as in MS/MS) is not feasible. For this reason, in our analysis, we focused on evaluating EI mass spectra collected solely in Q1 and Q3 scan modes. As shown in Figure 7, the isomers exhibit differences in the relative intensities of certain ions. For example, the ion at 221 *m*/*z* shows the following intensities: LSD (76.4%), MiPLA (79.5%) and LAMPA (81.2%). The ion at 207 *m*/*z* has intensities of LSD (43.9%), MiPLA (47.8%) and LAMPA (50.6%), while the ion at 181 *m*/*z* shows LSD (54.4%), MiPLA (40.7%) and LAMPA (60.4%). In the case of the iminium ion at 72 *m*/*z*, notable differences were also observed: LSD (35.5%), MiPLA (41.0%) and LAMPA (18.0%). For other isomers analyzed in this paper, analogous differences are noticeable, as presented in Figure 7. Compilation of all the MS data and corresponding ratios for various analogs presented in this paper may provide detailed MS data to facilitate comparison with results from samples containing unknown compounds. Often, subtle differences are not apparent in the spectra alone, and this approach can help differentiate, e.g., *iso*-analogs from the standard forms. Such modifications have already been observed in the drug market, as seen in compounds like fluorobutyrylfentanyl (FBF) and fluoroisobutyrylfentanyl (FiBF) [18] or α-pyrrolidinohexiophenone (α-PHP) and α-pyrrolidinoisohexiophenone (α-PiHP) [19].

### 2.2. Chromatographic Conditions and Separation of Structural Isomers

In the presented paper, a selective GC–MS method was established, enabling the separation of three sets of structural isomers (LSD, MiPLA and LAMPA; 1P-LSD and 1P-MiPLA; and 1cP-LSD and 1cP-MiPLA). Furthermore, the described chromatographic conditions allowed for the analysis of all 13 compounds in a single run. The elution order of the determined compounds was as follows: LSD (323 *m*/*z*; 27.860 min), MiPLA (323 *m*/*z*; 28.015 min), LAMPA (323 *m*/*z*; 28.205 min), AL-LAD (349 *m*/*z*; 28.560 min), LSZ (335 *m*/*z*; 28.660 min), 2-Br-LSD (401 *m*/*z*; 29.135 min), ALD-52 (365 *m*/*z*; 29.525 min), 1P-LSD (379 *m*/*z*; 30.195 min); 1P-MiPLA (379 *m*/*z*; 30.370 min), 1B-LSD (393 *m*/*z*; 30.915 min), 1V-LSD (407 *m*/*z*; 32.015 min), 1cP-LSD (391 *m*/*z*; 32.100 min) and 1cP-MiPLA (391 *m*/*z*; 32.345 min).

To improve sensitivity [20] or separation [17], derivatization techniques based on silylation are often used. However, this approach has considerable limitations in the context of the current drug situation, as the *N*1 atom is subject to derivatization (where a hydrogen atom is replaced by a silyl moiety). Increasingly, analogs with an amide moiety attached at *N*1 are being encountered, making it impossible to substitute these atoms with a derivatizing agent. For this reason, when analyzing unknown compounds in seized drug samples, derivatization is not recommended as a method of sample preparation.

**Figure 8:**
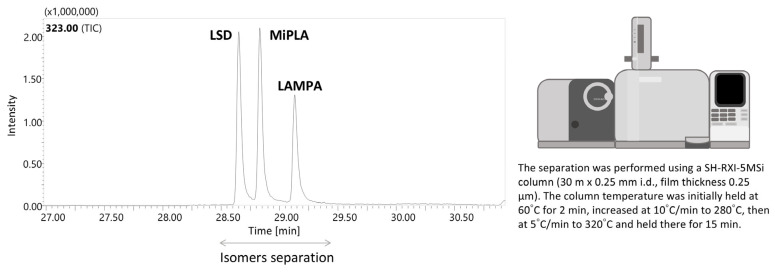
Additional chromatographic method dedicated for isomers separation (LSD, MiPLA and LAMPA).

The separation method presented in this publication enables the identification of the specific isomer contained in a sample. While it is unlikely that all three isomers (LSD, MiPLA and LAMPA) or two isomers (e.g., 1cP-LSD and 1cP-MiPLA; 1P-LSD and 1P-MiPLA) would be present simultaneously in a single sample, the method can be adapted to such cases. By modifying the gradient, it is possible to adjust the separation process for specific isomer groups. Initially, the chromatographic method provided clear separation of all isomer groups. However, to enhance the separation of LSD and MiPLA and to minimize peak tailing, the chromatographic gradient was optimized to more effectively resolve these compounds. Specifically, the temperature ramp rate was reduced from 10 °C/min to 5 °C/min at 280 °C, enabling the complete separation of all three compounds (Figure 8).

While GC is a gold standard for effective NPS determination and is widely used for toxicological analysis of evidence collected in crime scenes, there are several limitations when it comes to the differentiation of various designer LSD analogs. These limitations stem from both the inherent physicochemical properties of the compounds and the inherent constraints of the gas chromatography technique. The separation of LSD analogs may be influenced by the choice of chromatographic columns, temperature gradients, and carrier gas flow rates.

LSD and its analogs often exhibit highly similar molecular structures, which can pose challenges in their differentiation. Moreover, the growing availability of novel psychoactive substances (NPSs) in the illicit market has led to the introduction of various isomeric forms of these compounds. These isomers are frequently marketed as a means to bypass existing legal regulations, further complicating the identification and analysis of these substances. This structural similarity can lead to the co-elution of some isomers, making their complete separation challenging when using standard, short-gradient GC methods. In the presented paper, the gradient profile was initially slow, with a total run time ranging from 43 to 47 min (for the second method dedicated to LSD, MiPLA and LAMPA). This approach allowed for excellent separation of all 13 compounds in a single run. Furthermore, this method has the potential to be applied to newly emerging analogs, including isomeric compounds, that may appear in the future.

In the context of GC methods, the thermal stability of certain compounds is a critical factor to consider. The high temperatures required at the injection port can present challenges for thermally labile compounds. However, aside from the instability of designer LSD analogs when using methanol as a solvent, no significant thermal effects were observed. Notably, even analogs containing *N*1-moieties can be effectively analyzed using the proposed method.

### 2.3. The Chemical Aspect of Solvent Effects on GC–MS Analysis

In forensic toxicology, gas chromatography–mass spectrometry (GC–MS) is a standard analytical technique used for the identification of substances, including LSD analogs, in seized drug samples. These samples may contain mixtures of substances, including active ingredients (e.g., LSD analogs) cutting agents, fillers and other adulterants. The solvent’s ability to dissolve LSD analogs and separate them from these other substances is important for achieving clear, reliable results. However, when considering the use of solvents in the analysis of LSD analogs with the use of GC–MS, several additional factors must be taken into account.

First and foremost, volatility is crucial, as the solvent must evaporate efficiently during the injection process. Solvents that leave residues or do not evaporate quickly can interfere with mass spectrometry detection, potentially causing ion suppression or signal noise. Thus, all solvents discussed in this paper are volatile, ranging from very volatile (e.g., diethyl ether and dichloromethane), through slightly less volatile (e.g., tert-butyl methyl ether, acetone, methanol, ethanol, ethyl acetate and hexane), to even less volatile (e.g., acetonitrile, 2-propanol and isooctane). However, they are still suitable for GC–MS analysis as their boiling points [21,22,23,24] are significantly lower than the temperature in the injection port allowing for effective evaporation.

**Figure 9:**
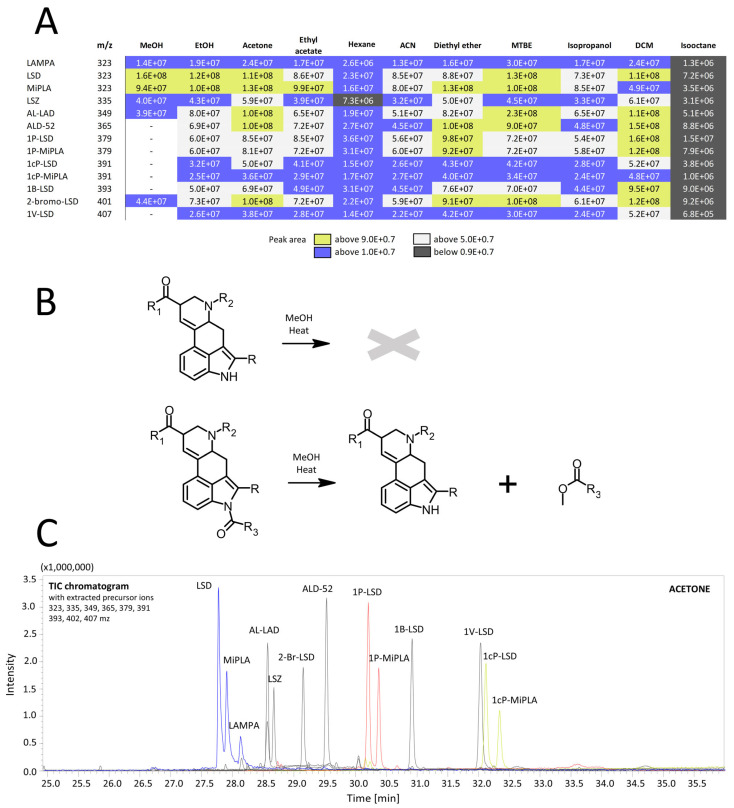
Results obtained in different solvents (**A**), mechanism of hydrolysis reaction (**B**) and chromatogram of all analyzed compounds in acetone (**C**).

Another very important aspect to consider is the solubility of analytes in the solvent, as it directly impacts the concentration of analytes in the sample solution. If LSD analogs do not dissolve well in the solvent, the effective concentration of the analytes decreases, leading to poor signal intensity during GC–MS analysis. Given that LSD and its analogs are typically taken in low-dose formulations, it is expected that, the sensitivity of the developed analytical method will be crucial in terms of forensic investigations. Considering LSD analogs in this aspect, it is important to determine their polarity and compare it to the polarity of the solvents used. The logP value, which describes the partition coefficient between two immiscible solvents (typically octanol and water), for LSD analogs fall between 1.601 and 3.191 values (Figure 1), which makes them low to moderate polar compounds. This potentially leads to the supposition that they should dissolve best in low-polarity solvents, which would, in turn, lead to better signal intensity in GC–MS analysis. Considering the results of analyte signal areas obtained from GC–MS analysis (Figure 9A), it can be concluded that they coincide with theoretical expectations. The worst results were obtained using hexane and isooctane, which are non-polar solvents [25]. This may be due to the presence of polar features in the LSD analogs’ structures, such heteroatoms (e.g., nitrogen) or functional groups such as a carbonyl group, which make them poorly soluble in non-polar solvents. On the other hand, the best results were obtained using moderately polar and low-polar solvents, such as dichloromethane, tert-butyl methyl ether, diethyl ether, acetone and ethyl acetate [25]. This coincides with our conjecture, since their degree of polarity is most similar to that of LSD analogs, which makes these solvents able to interact efficiently with both polar and non-polar fragments of analytes. Figure 9A shows that dissolving LSD analogs in 2-propanol also yielded unsatisfactory results, despite its moderately polar nature. Such a result may be due to the fact that 2-propanol (as a polar and protic solvent) forms hydrogen bonds with the functional groups of the LSD analogs limiting interactions between LSD molecules themselves and creating a less favorable solvation environment. To the best of our knowledge, no other study has examined this many solvents in relation to signal intensity in GC–MS analysis, which directly affects the sensitivity of the analytical method. Typically, studies on LSD analog analysis by GC–MS focus on dissolving the analytes in methanol or acetonitrile [11,17]. Zhang et al. [12] analyzed two LSD analogs (ALD-52 and 1P-LSD) using acetonitrile, methanol, ethanol, isopropyl alcohol, ethyl acetate and acetone but in a slightly different context. The authors concluded that non-alcoholic solvents should be used for LSD analog analysis, which aligns with the findings of this study.

This issue is also related to the phenomenon of deacylation of LSD analogs in alcoholic solutions, which can be a significant problem in toxicological analysis of physical evidence when it is necessary to determine which specific analog is present, as it has legal and judicial implications. Alcohols like methanol and ethanol cause alcoholysis of LSD analogs containing an amide group as a substituent at N1. According to the mechanism proposed by Zhang et al. [12], alcohols with strong nucleophilic character attach to the carbon of the carbonyl group in the amide moiety, leading to the breaking of the amide bond, the detachment of the corresponding ester molecule (whose structure depends on the alkyl chain of the substituent at N1) and the formation of the LSD (in the case of ALD-52, 1P-LSD, 1cP-LSD, 1B-LSD and 1V-LSD) or MiPLA (in the case of 1P-MiPLA and 1cP-MiPLA) structure (Figure 9B). The results presented in this paper show that when dissolving analytes in methanol, no signal is observed from analogs capable of undergoing deacylation, namely ALD-52, 1P-LSD, 1P-MiPLA, 1cP-LSD, 1cP-MiPLA, 1B-LSD and 1V-LSD. In contrast, in other solvents tested, which are less nucleophilic, all the LSD analogs were detected. Figure 9C shows an example chromatogram obtained after dissolving a mixture of 13 LSD analogs in acetone. In the work by Zhang et al. [12] the dependence of the signal intensity profile of ALD-52, 1P-LSD and the resulting LSD on the solvent used was noted. These results coincide with those observed in the presented work, where, when using methanol, no signal is observed from compounds that can convert to LSD by deacylation. In contrast, as in the work by Zhang et al., solvents such as acetone, ethyl acetate and acetonitrile provided the possibility of observing signals from all analytes tested on the chromatogram. They also point out the partial deacylation under the influence of ethanol and its absence in the case of 2-propanol, which is also observed in the presented work where in the latter two solvents, all the analogs were able to be detected. In the case of compounds undergoing alcoholysis with MiPLA formation, Tanaka et al. [11] observed no deacylation of 1cP-MiPLA using acetonitrile solution, which matches the present findings, where all compounds were present in the chromatogram. However, in methanol, no signals from 1cP-MiPLA or 1P-MiPLA were detected. In their paper, however, Tanaka et al. [11] mention that the 1V-LSD analog underwent partial deacylation to LSD, but this was not observed for 1cP-AL-LAD. This suggests that additional factors may influence the hydrolysis of LSD analogs in GC–MS analysis. One such factor could be the concentration of the compound being analyzed. At higher concentrations, the compound’s molecules tend to cluster, reducing degradation, while at lower concentrations, they are more solvated, leading to possible increased degradation. This was confirmed by Zhang et al. [12] in their experiment in methanol and acetonitrile. Some other factors that other authors have tested for reducing the degradation of new psychoactive substances during GC–MS analysis included different split ratios [13] or injection temperatures [26].

The results of this study underscore the importance of selecting the appropriate solvent for chromatographic analysis, especially in forensic toxicology, where identifying the specific analog present in a sample is crucial. The legal consequences of possessing, being under the influence of or dealing with specific LSD analogs necessitate accurate identification of the compound in seized drug samples. Using methanol as a solvent could lead to erroneous results, detecting LSD or MiPLA from the decomposition of another analog. In criminal investigations, where linking a suspect, dealer or deceased person to the same substance is essential; relying on methanol as a solvent could mislead the analysis, detecting only the degradation product rather than the compound actually present in the sample.

To summarize the issue of selecting the solvent used for GC–MS analysis of LSD analogs, attention should be paid to its polarity and the polarity of the solvent, as well as its possible effect on the deacylation of the analyzed compounds. Of the solvents tested in this study, diethyl ether and tert-butyl methyl ether, acetone and dichloromethane appear to be the best ones. However, the first two solvents, despite the fact that they give satisfactory results (high sensitivity of the assay and the possibility of detection of all analogs), are problematic in use as, due to their high volatility, there is a possibility of loss in the extract when transferred to the vial. Dichloromethane, on the other hand, is more toxic than, ethyl acetate or acetone, which also allow for the detection of all analyzed compounds with satisfactory sensitivity, making them better suited for forensic toxicology analysis.

### 2.4. UV Spectra Analysis

**Figure 10:**
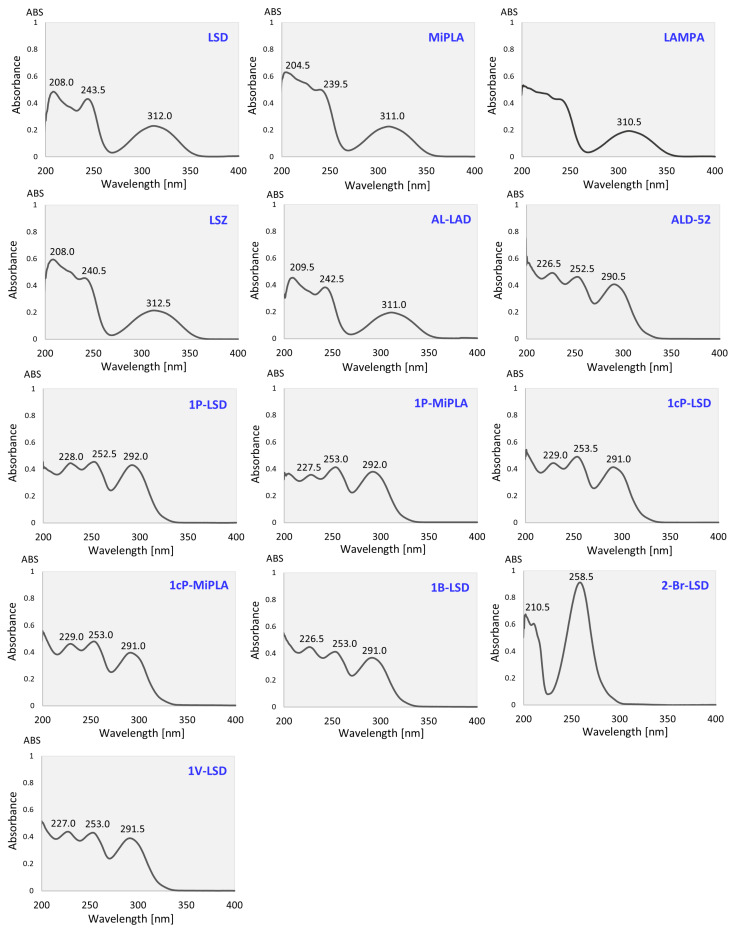
UV spectra of compounds analyzed in methanol.

In addition to the most commonly used GC–MS technique, UV spectroscopy can also be utilized to analyze seized drug samples. As a cheaper method, it can be used for screening analysis of evidence containing LSD analogs. Spectra of all analyzed 13 compounds obtained in methanol are shown in Figure 10. At the beginning, it should be noted that in a solution, LSD analogs do not undergo alcoholysis until they are heated and the methanol is vaporized in the MS injection port [12]. Hence, it can be assumed that the observed UV spectra are those of the LSD analogs under study.

The compounds analyzed in this paper were initially at the concentration of 100 μg/mL; however, after evaporation and dilution in a cuvette for UV measurements, their final concentration was about 8 μg/mL. Satisfactory absorbance was obtained for all the compounds, with distinguishable absorption maxima, which indicates that the method can be successfully applied to the analysis of seized drugs, such as blotter papers, where doses typically range from 50 to 200 μg [27].

As can be observed, the UV spectra obtained can be divided into three groups, which also strictly relate to the structure of the analyzed compounds. LSD and its analogs without a substituent at the N1 position (MiPLA, LAMPA, LSZ and AL-LAD) are characterized by similar spectra with absorption maxima at around 205–209 nm, 240–243 nm and 310–312 nm. It is worth noting that of the mentioned compounds, the spectrum of LAMPA is characterized by the least isolated maxima at lower wavelengths. Adding a substituent at the N1 position results in a change in the UV spectrum. A bathochromic shift (toward longer wavelengths) is observed in the first band and is characterized by a maximum at about 226–227 nm for analogs with a linear aliphatic chain in the substituent (ALD-52, 1P-LSD, 1P-MiPLA, 1B-LSD and 1V-LSD) or 229 nm for analogs with a cyclic chain in the substituent (1cP-LSD and 1cP-MiPLA). The second band also shifts toward longer wavelengths and exhibits a maximum at around 253 nm for all analogs in this group. The third band, on the other hand, undergoes a hypsochromic shift (toward lower wavelengths) and exhibits an absorption maximum at around 291–292 nm for all compounds with a substituent at N1. The only UV spectrum that stands out firmly from the others is the 2-Br-LSD spectrum, which exhibits a maximum at around 210 nm (a slight hypsochromic effect relative to the first band of unsubstituted analogs at N1) and a marked maximum at 258.5 nm. Such a large difference from the rest of the spectra may be due to the effect of bromine as a strongly electro-negative substituent on electron distribution and energy and thus on electron transitions in UV spectroscopy, which is also observed in other classes of new psychoactive substances, e.g., synthetic cathinones [28].

The literature on UV spectra of LSD analogs is very scarce and includes mainly papers where UV spectroscopy was used as a diode array detector (DAD) in liquid chromatography analysis. Other authors, as in the presented study, observed three absorption bands for 1P-LSD [29], LSZ [30] and 1B-LSD [31]. Brandt et al. [30] reported an AL-LAD spectrum that exhibits two maxima, one of which is poorly distinguished, while in the presented work, three distinct absorption maxima can be observed. Both this and the fact that the spectra observed by the cited authors showed shifted maxima by a few nm compared to this work are probably due to the fact that the mobile phase in their analyses was acetonitrile and water (with the addition of buffer). Depending on the retention time of a given analyte, the composition of the mobile phase (acetonitrile–water mixture) will vary, making it difficult to determine unambiguously in which solution specific compounds were analyzed. The presence of water and additives can affect the position of the absorption bands. For instance, in the case of LSZ [30] and 1P-LSD [29], the first two bands, and in the case of AL-LAD [30], only one of them (as the other probably shifted out of the analyzed range) show a bathochromic effect compared to the bands observed in pure acetonitrile in the presented work. For AL-LAD and LSZ [30], the band located at the highest wavelengths shows a maximum at similar values as in this work, while for 1P-LSD [29] and 1B-LSD [31], it is shifted toward longer wavelengths. It should be mentioned, however, that analysis of other LSD analogs, also using the DAD detector, has shown that they also have maxima at similar wavelengths to those mentioned above, i.e., at around 226 nm, 253 nm and 310 nm (in the case of ETH-LAD and 1P-ETH-LAD [32]) or with a shift in the position of the last band to 294 nm (in the case of 1DD-LSD [33]). LSM-775, due to its additional cyclic moiety, has the first two bands shifted toward lower wavelengths (202 and 223 nm), while the third band is shifted toward higher wavelengths (313 nm) [34]. 1T-LSD, which contains a moiety with a sulfur atom also exhibits different absorption maxima: at 235 and 293 nm when the spectrum is recorded in pure acetonitrile [11]; however, it is worth mentioning that it was a seized drug, not of an analytical standard, that was examined, so other additives might have influenced the position of the maxima.

The examples described here show that while the appearance of the spectrum and the position of the absorption bands can be of some help in identifying a given group of LSD analogs, it is not a method without drawbacks. It is heavily influenced by the type of solvent used, which affects the position of the absorption maxima. What is more problematic is the analysis of mixtures of analogs or even contaminated paper with another compound that also has chromophores because there will be interference in the analysis resulting from no separation method. Sometimes it happens that, at the same time, there are two substances in one evidence because of contamination in clandestine laboratories and drug manufactures or by an inadequate synthesis pathway. Thus, in forensic toxicology, this method can serve as a screening method, but it would be necessary each time to compare the obtained spectrum with that of the standard, which, however, does not exclude the need to confirm the result with a more reliable method like GC–MS.

### 2.5. Strategies for LSD Analogs Analysis in Practical Settings

**Figure 11:**
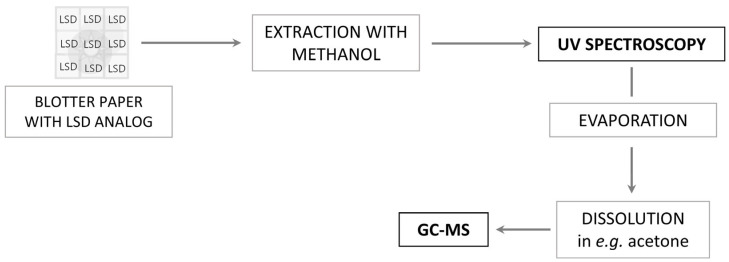
Summarization of the pre-analytical strategy for examination of authentic blotter papers with the use of two techniques from one sample.

Taking all the aspects described in the above paragraphs into account, it is possible to present a general scheme of practice that could be implemented in a forensic laboratory for the analysis of physical evidence potentially containing LSD analogs (Figure 11). The resulting evidence could be screened using UV spectrophotometry by placing, for example, a paper blotter in methanol and subjecting it to ultrasound for 15 min. The methanol solution can then be directly transferred to a cuvette for UV measurements and have its spectrum recorded, which should then be compared with the spectrum of the analytical standard recorded under identical conditions.

The next step should be to use the GC–MS/MS technique, where the physical evidence’s solution used in the first step should be evaporated and then the dry residues should be dissolved in the chosen solvent. Based on the results presented in this paper, the authors would recommend using ethyl acetate, diethyl ether, tert-butyl methyl ether, acetone or dichloromethane. The final choice should depend on what analog is potentially present in the evidence under study (selection of the appropriate solvent polarity) and the availability of the described solvents in a given laboratory. In the final step, GC–MS/MS analysis should make it possible to determine which analog one is dealing with, including distinguishing isomers, which can be carried out by analyzing ion ratios on the EI spectrum. Such a comprehensive measure will enable in-depth analysis of physical evidence for LSD analogs for forensic toxicology purposes.

## 3. Materials and Methods

### 3.1. Chemicals

Acetonitrile (Chromasolv^®^ LC–MS), acetone (Chromasolv^®^ LC–MS), methanol (Chromasolv^®^ LC–MS), 2-propanol (Chromasolv^®^ LC–MS), ethanol (LiChrosolv^®^ LC–MS), isooctane (LiChrosolv^®^ LC–MS), diethyl ether (Chromasolv^®^ LC–MS), dichloromethane (Chromasolv^®^ LC–MS), ethyl acetate, n-hexane and tert-butyl methyl ether were purchased from Sigma-Aldrich (Steinheim, Germany). Standard solutions of: 1V-LSD, LSZ, AL-LAD, 1cP-LSD, MiPLA, 1B-LSD, ALD-52, 1P-MiPLA, 1P-LSD, 2-Bromo-LSD and 1cP-MiPLA (each substance in a concentration of 100 μg/mL in acetonitrile) and LAMPA (1 mg/mL in acetonitrile) were obtained from Cayman Chemical (Ann Arbor, MI, USA). LSD (1 mg/mL in acetonitrile) was purchased from Cerilliant (Round Rock, TX, USA).

LSD and LAMPA standards at a stock concentration of 1 mg/mL were diluted 10 times with acetonitrile, which served to prepare a working solution in the concentration of 100 μg/mL. The stock solutions and standard solutions were stored at −20 °C.

### 3.2. Instrumentation

The analyses were performed using a gas chromatograph (GC, Shimadzu QP2010; Kyoto, Japan) operated with an autosampler AOC-20s and autoinjector AOC-20i (Shimadzu, Milan, Italy). The separation was performed using an SH-RXI-5MSi column (30 m × 0.25 mm i.d., film thickness 0.25 µm; Shimadzu, Bellefonte, PA, USA). The parameters for GC and MS were similar to those presented earlier [35], with slight modifications to the gradient profile to tailor the method for the large number of isomers. The column temperature was initially held at 60 °C for 2 min, increased at 10 °C/min to 320 °C and held there for 15 min. Helium (purity 6.0, Messer, Gumpoldskirchen, Austria) was used as a carrier gas at a flowrate of 1.56 mL/min. The syringe size was 10 µL. The injection volume was 1 μL. A splitless injection mode was applied with sampling time of 1.0 min. The injector temperature was 260 °C. The injector was set to auto cleaning by pre-injecting ethyl acetate and acetonitrile. The total run time was 43 min. Detection was achieved using a triple quadrupole mass spectrometer (QqQ, Shimadzu TQ8040, Kyoto, Japan). The spectrometer was equipped with an electron ionization (EI) source; mass spectrometer was operated in a Q1 and Q3 scan mode in the range 20–600 *m*/*z*. The following MS parameters were fixed: ion source temperature, 200 °C; interface temperature, 280 °C; electron ionization energy, 70 eV; and the detector voltage set at 0.4 kV. A clear glass conical base with polyspring inserts (6 × 29 mm) were used for the GC–MS/MS analysis.

A Shimadzu UV-1900i UV/VIS (Canby, OR, USA) spectrophotometer equipped with a quartz cell (Starna Scientific, Pfungstadt, Germany) with 10 mm path length were used. Measurement of absorbance spectra was carried out in the UV range 200–400 nm with 0.5 nm resolution. Prior to any compound analysis, the methanol blank was analyzed. The spectrometer was controlled using LabSolutions UV-Vis version 1.03 software (2018 Shimadzu Corporation, Canby, OR, USA), which allowed to collect necessary data.

### 3.3. Sample Preparation

GC-EI-MS: A volume of 10 μL of each analytical standard of analyzed compounds at a concentration of 100 μg/mL was combined together in a 2 mL Eppendorf-type tube. The mixture prepared in this way was then evaporated under a stream of an inert nitrogen at 40 °C. A total of 50 μL of the appropriate solvent was then added to the dry residues, and everything was vortex mixed for 10 s and transferred to the glass GC vial.

UV: A volume of 50 μL of each analytical standard (at a concentration of 100 μg/mL) was separately placed in a 2 mL Eppendorf-type tube and evaporated under a stream of inert nitrogen at 40 °C. A total of 600 μL of methanol was then added to the dry residues and vortex mixed for 10 s, and the mixture was then transferred to a quartz cuvette for the UV analysis.

## 4. Conclusions

The work presented here demonstrates how many important issues need to be taken into account when analyzing new psychoactive substances from the LSD analog group using the most commonly used techniques for this purpose, such as GC–MS and UV spectroscopy. The need for good chromatographic separation of isomers, careful consideration of ion ratios in mass spectrometry, as well as the selection of an appropriate solvent to avoid hydrolysis of analytes will enable the correct determination of newly emerging compounds on the market. What is more, it could be applied to the results obtained for forensic purposes such as linking the material evidence in question to the person who potentially ingested it or to the person responsible for dealing and distributing illegal substances.

## Data Availability

The original contributions presented in the study are included in the article, further inquiries can be directed to the corresponding author.

## Abbreviations

1B-LSD: 1-Butanoyl-lysergic acid *N*,*N*-diethylamide; 1cP-AL-LAD: 1-cyclopropanecarboxyl-*N*-allylnorlysergic acid *N*,*N*-diethylamide; 1cP-LSD: 1-Cyclopropionyl-lysergic acid *N*,*N*-diethylamide; 1cP-MiPLA: 1-Cyclopropionyl-lysergic acid methylisopropylamide; 1DD-LSD: 1-Dodecanoyl-lysergic acid *N*,*N*-diethylamide; 1P-ETH-LAD: *N*-Propionyl-6-ethyl-6-nor-lysergic acid diethylamide; 1P-LSD: 1-Propionyl-lysergic acid *N*,*N*-diethylamide; 1P-MiPLA: 1-Propinoyl-lysergic acid methylisopropylamide; 1T-LSD: 1-(thiophene-2-carbonyl)-*N*,*N*-diethyllysergamide; 1V-LSD: 1-Valeroyl-lysergic acid *N*,*N*-diethylamide; ALD-52 (1A-LSD): 1-Acetyl-lysergic acid *N*,*N*-diethylamide; AL-LAD: *N*-Allylnorlysergic acid *N*,*N*-diethylamide; ETH-LAD: *N*-Ethylnorlysergic acid *N*,*N*-diethylamide; LAMPA: Lysergic acid methylpropylamide; LSD: *N*,*N*-Diethyllysergamide; LSM-775: Lysergic acid morpholide; LSZ: Lysergic acid 2,4-dimethylazetidide; MiPLA: Lysergic acid methylisopropylamide.
